# Effective control in MSI-high unresectable intrahepatic cholangiocarcinoma treated with gemcitabine/cisplatin/durvalumab and proton beam therapy: a case report

**DOI:** 10.3389/fimmu.2025.1685944

**Published:** 2025-10-23

**Authors:** Takuto Nosaka, Hironori Naito, Yosuke Murata, Yu Akazawa, Tomoko Tanaka, Kazuto Takahashi, Tatsushi Naito, Masahiro Ohtani, Yoshiaki Imamura, Yasunari Nakamoto

**Affiliations:** ^1^ Second Department of Internal Medicine, Faculty of Medical Sciences, University of Fukui, Fukui, Japan; ^2^ Division of Diagnostic Pathology/Surgical Pathology, University of Fukui Hospital, Fukui, Japan

**Keywords:** intrahepatic cholangiocarcinoma, immune checkpoint inhibitor, proton beam therapy, vascular invasion, microsatellite instability

## Abstract

Systemic chemotherapy is the standard treatment for unresectable intrahepatic cholangiocarcinoma (iCCA); however, its efficacy remains limited. We report a clinically valuable case of a 57-year-old woman with advanced unresectable iCCA characterized by high microsatellite instability (MSI-high) and major vascular invasion. The patient presented with purpura and was diagnosed with MSI-high iCCA with intravascular tumor extension reaching the right atrium and regional lymph node metastases. Combination therapy with gemcitabine, cisplatin, and durvalumab (GCD therapy) was initiated, resulting in a 56% reduction in tumor size after eight cycles. To enhance local tumor control, proton beamtherapy (3.3 Gy × 22 fractions) was added, which was completed without adverse events. The patient subsequently received durvalumab maintenance therapy, followed by pembrolizumab. Although there was minimal growth of lymph node and pulmonary metastases, no regrowth of the intrahepatic primary tumor was observed for 18 months after PBT. This case illustrates the potential clinical value of combining GCD therapy with proton beam therapy for MSI-high, unresectable iCCA with major vascular invasion. The combination achieved systemic disease control and durable local control without significant toxicity.

## 1 Introduction

Cholangiocarcinoma is anatomically classified into intrahepatic (iCCA) and extrahepatic subtypes (perihilar and distal). iCCA is the second most common subtype of primary liver cancer, accounting for approximately 15% of all primary liver cancers worldwide, and its incidence is increasing (1). Because the disease often progresses asymptomatically, a large proportion of patients are unresectable at diagnosis, and recurrence is common; consequently, long-term outcomes remain poor (2). For unresectable iCCA, based on the TOPAZ-1 trial, gemcitabine, cisplatin, and durvalumab (GCD) has been established as the standard first-line regimen (3); however, systemic chemotherapy alone may be insufficient to prevent local progression and liver-related events in a subset of patients (4). Accordingly, the optimal integration of systemic and local therapies remains a key clinical challenge. At the molecular level, a subset of iCCA harbors high microsatellite instability (MSI-high) (5), and such tumors have demonstrated increased sensitivity to immune checkpoint inhibitors (ICIs) (6).

Among high-precision radiotherapy modalities, proton beam therapy (PBT) provides superior dose conformality, enabling high-dose irradiation while sparing organs at risk (OARs) such as the hepatic parenchyma, biliary tract, and gastrointestinal structures; long-term survival has been reported in unresectable iCCA (7–9). Furthermore, because radiotherapy can modulate the tumor immune microenvironment, combining it with ICIs is expected to achieve both systemic disease control and durable local control (10). However, much of the evidence derives from nonrandomized studies and registry cohorts, and there remains scope for prospective optimization of combination strategies (3, 11).

Here, we report a case of unresectable MSI-high iCCA with major vascular invasion, in which successful local control was achieved by combining GCD therapy with subsequent PBT.

## 2 Case description

**Figure 1:**
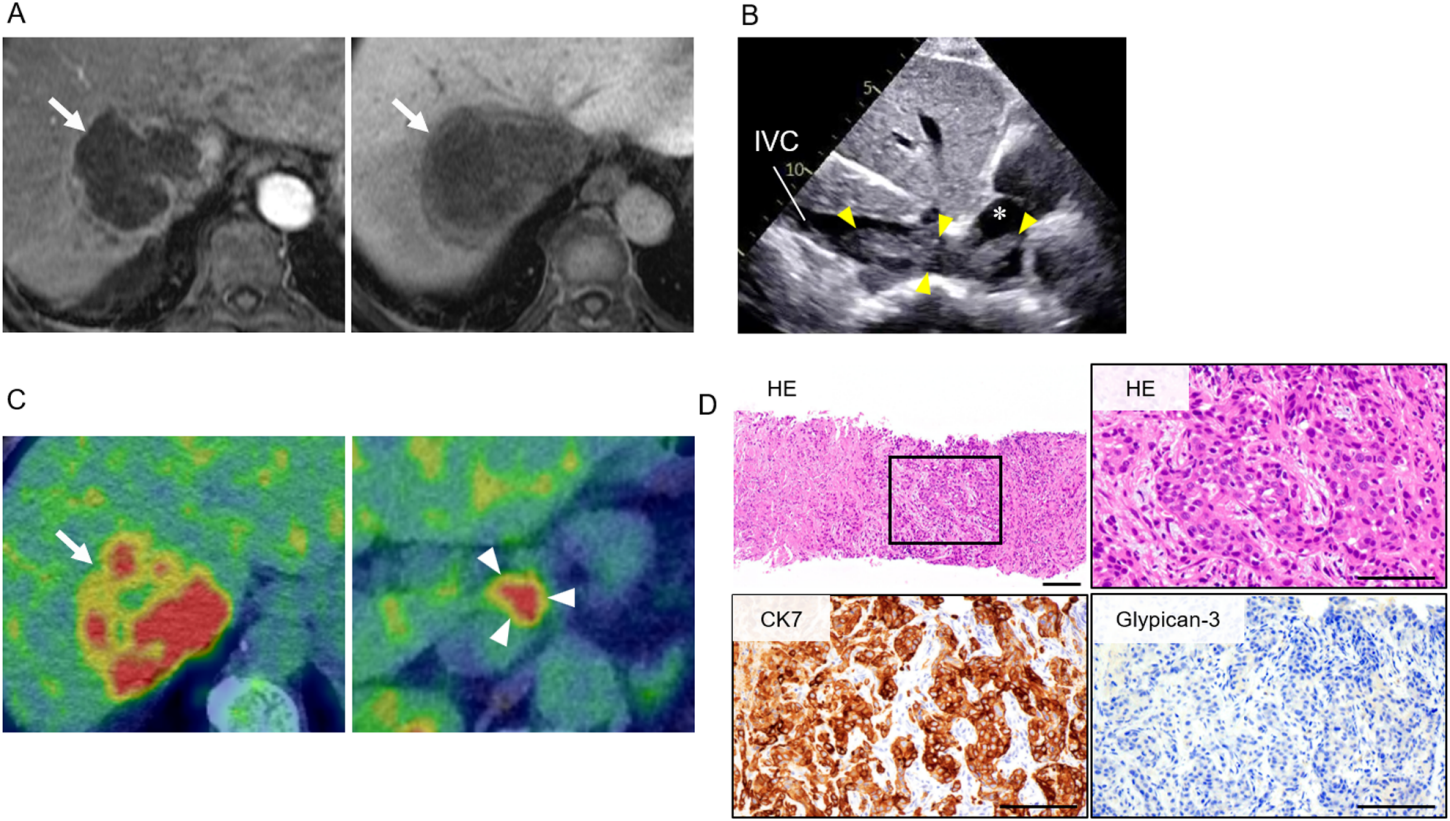
Imaging findings and liver tumor biopsy of unresectable intrahepatic cholangiocarcinoma (iCCA). **(A)** Gadoxetic acid-enhanced magnetic resonance imaging (EOB-MRI) images. Left: arterial phase; Right: hepatobiliary phase. The intrahepatic tumor is indicated by white arrows. **(B)** Transabdominal ultrasound image. Tumor invasion into the inferior vena cava (IVC) is observed (yellow arrowhead), with partial extension into the right atrium (white asterisk). **(C)** Fluorodeoxyglucose positron emission tomography/computed tomography (FDG PET/CT) findings. Left: High uptake in the intrahepatic tumor (arrow). Right: High uptake in the metastatic hilar lymph node (arrowhead). **(D)** Histopathological findings of the liver tumor obtained by percutaneous needle biopsy. From left to right: Hematoxylin and eosin (H&E) staining (low magnification), H&E staining (high magnification), immunohistochemistry for cytokeratin 7 (CK7), and Glypican-3. Bar, 100 µm.

**Figure 2:**
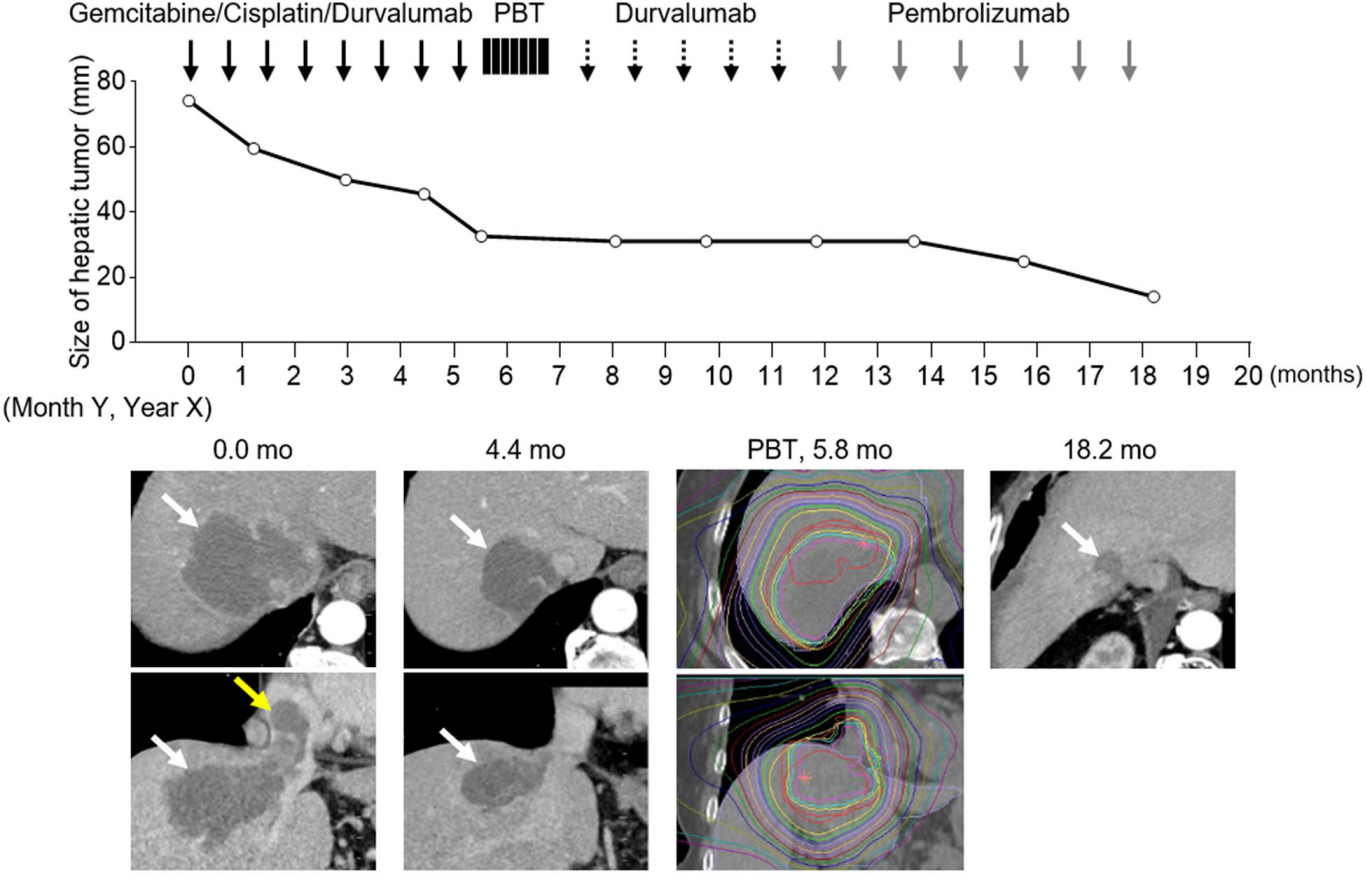
Clinical course of the patient. Treatment was initiated in Month Y, Year X (Day 0). Contrast-enhanced computed tomography (CT) images show the intrahepatic lesion (white arrows, white dot lines), intravascular invasion extending into the right atrium (yellow arrows), and the dose distribution of proton beam therapy (PBT).

A 57-year-old woman noticed purpura on her left buttock and limbs in Month Y–1, Year X, and visited a nearby clinic. She had no significant past medical history. Laboratory tests revealed anemia and decreased fibrinogen levels, raising suspicion of fibrinolytic-dominant disseminated intravascular coagulation (DIC) (**Table 1**). The calculated DIC score according to the International Society on Thrombosis and Haemostasis (ISTH) criteria was 5, confirming the diagnosis of DIC. Gadoxetic acid-enhanced magnetic resonance imaging (EOB-MRI) and transabdominal ultrasound revealed a 77-mm mass in the right hepatic lobe (**Figure 1A**), with tumor invasion extending from the inferior vena cava into the right atrium (**Figure 1B**). She was referred and admitted to our department for further evaluation. Fluorodeoxyglucose positron emission tomography/CT (FDG PET/CT) demonstrated intense uptake in the hepatic tumor, with a maximum standardized uptake value (SUVmax) of 13.3, as well as in the hilar lymph nodes (**Figure 1C**). A percutaneous biopsy of the liver tumor demonstrated adenocarcinoma with CK7-positive and Glypican-3-negative immunostaining (**Figure 1D**), consistent with intrahepatic cholangiocarcinoma. Given the patient’s advanced malignancy and laboratory findings, a tumor-related DIC was suspected. Comprehensive genomic profiling using the FoundationOne CDx panel revealed high microsatellite instability (MSI-high). Combination chemotherapy with gemcitabine, cisplatin, and durvalumab (GCD therapy) was initiated in Month Y, Year X, resulting in a 56% reduction in tumor size after eight cycles, according to the Response Evaluation Criteria in Solid Tumors (RECIST 1.1) (**Figure 2**). To achieve further local control of the intrahepatic lesion, proton beam therapy was initiated at 5.8 months following treatment initiation. The patient was immobilized individually in the supine position, and respiratory motion of the tumor was assessed using respiratory-gated four-dimensional computed tomography (4D-CT). Treatment planning ensured dose coverage of the planning target volume (PTV; V95%) while adhering to dose constraints for OARs. The prescribed regimen consisted of 3.3 Gy (RBE) per fraction, once daily, five days per week, for a total of 22 fractions (72.6 Gy [RBE]). Irradiation was delivered under respiratory gating, with image-guided position verification performed before each session. The patient was subsequently transitioned to maintenance therapy with durvalumab. At 12 months after treatment initiation, new pulmonary metastases were detected and subsequently showed progression, prompting a switch to pembrolizumab. Following ICI-based chemotherapy combined with PBT, the ISTH score improved to 0. No treatment-related toxicities were observed (Common Terminology Criteria for Adverse Events [CTCAE], version 5.0). At 18 months, the intrahepatic tumor remained stable without evidence of progression, and local control was maintained. At the latest follow-up, 26 months after treatment initiation, the patient remains alive.

**Table 1:** Laboratory examinations.

WBCs	5, 800	/µL	BUN	12	mg/dL
RBCs	283×10^4^	/µL	Creatinine	0.43	mg/dL
Hemoglobin	8.8	g/dL	Total protein	6.7	g/dL
Hematocrit	28.3	%	Albumin	3.5	g/dL
Platelets	14.2×10^4^	/µL	Total bilirubin	0.4	mg/dL
			Aspartate aminotransferase	54	U/L
PT-INR	1.25		Alanine aminotransferase	55	U/L
APTT	33.5	sec	LDH	1962	U/L
Fibinogen	<50	mg/dL	ALP	718	U/L
FDP	78.2	μg/mL	γGTP	73	U/L
D-dimer	22.4	μg/mL	CRP	0.16	mg/dL
TAT	64.8	ng/mL	HbA1c	5.6	%
PIC	12.1	μg/mL			
CEA	1.8	ng/mL	HBsAg	–	
CA19-9	58.3	U/mL	HCVAb	–	
CA125	90.4	U/mL			
AFP	2.5	ng/mL			
DCP	32	mAU/mL			

WBCs, white blood cells; RBCs, red blood cells; PT-INR, prothrombin time-international normalized ratio; APTT, activated partial thromboplastin time; FDP, fibrinogen/fibrin degradation products; TAT, thrombin-antithrombin complex, PIC; plasmin-alpha2 plasmin inhibitor complex, BUN; blood urea nitrogen; LDH, lactate dehydrogenase, ALP; alkaline phosphatase; γGTP, gamma-glutamyl transpeptidase; CRP, C-reactive protein; HbA1c, glycated hemoglobin; CEA, carcinoembryonic antigen; CA19–9, cancer antigen 19–9; AFP, alpha-fetoprotein; DCP, des-gamma-carboxy prothrombin; HBsAg, hepatitis B virus surface antigen; HCVAb, anti-hepatitis C virus antibodies.

## 3 Discussion

We experienced a case of unresectable iCCA that demonstrated significant tumor shrinkage and systemic disease control following GCD therapy, with additional effective local control achieved through PBT. Because iCCA is an aggressive malignancy, few patients are eligible for surgical resection at the time of diagnosis; thus, systemic chemotherapy and radiotherapy are commonly employed (2). In the TOPAZ-1 trial (12, 13), the addition of an ICI to gemcitabine and cisplatin improved overall survival, establishing GCD therapy as the new standard first-line treatment (3). In this case, comprehensive genomic profiling of the tumor biopsy confirmed MSI-high status. The prevalence of MSI-high is reported to be only 0–2.1% in biliary tract cancers (14) and 4.7% in iCCA (5). MSI-high tumors are known to be highly sensitive to ICIs (6), which may have contributed to the favorable response to ICI-based therapy observed in this case.

Although chemotherapy remains the standard first-line treatment for unresectable iCCA, its efficacy is limited, underscoring the urgent need for combination strategies with effective local therapies (4). In patients with iCCA and extrahepatic metastases, radiotherapy to the primary liver tumor has been reported to significantly delay the progression of local disease, the onset of tumor-related liver failure, and death (15). Particle beam therapy offers excellent dose concentration and has the potential to achieve high local control rates (11). In unresectable iCCA, PBT has demonstrated 1-year local control rates ranging from 91.2% to 100%, along with favorable overall survival outcomes (11, 16). Several studies have demonstrated the efficacy of combining chemotherapy and radiotherapy in biliary tract cancers (17, 18). Additionally, a case report has shown a favorable therapeutic response to the combination of a PD-1 inhibitor and radiotherapy (19). In this case, GCD therapy was selected as the initial treatment, resulting in shrinkage of the primary tumor and a tendency toward reduction of lymph node metastases, although complete response was not achieved. To enhance local control, PBT was administered, which led to further improvement in local control without any adverse events. To our knowledge, this is the first reported case of unresectable iCCA treated with PBT following GCD therapy, highlighting its clinical significance.

In resectable iCCA, surgical resection is generally the first-line option (3); however, this patient was unresectable owing to major vascular invasion with tumor thrombus extending from the inferior vena cava to the right atrium and concomitant metastases. Although radiofrequency ablation (RFA) can yield favorable outcomes in small, localized tumors (20), its indications are limited for a 77-mm, vessel-adjacent lesion with tumor thrombus because the heat-sink effect and procedural complications increase the risk of incomplete ablation. By contrast, PBT offers superior dose conformality, allowing delivery of adequate tumoricidal doses while sparing the normal liver, biliary tract, and gastrointestinal organs (11). In this case, consolidation PBT after GCD achieved durable local control of the primary lesion without treatment-related adverse events. Accordingly, for advanced cases ineligible for resection or RFA, a sequential strategy of “systemic therapy first, followed by high-precision radiotherapy for local consolidation” may represent a practical option. As this is a single case, modality superiority cannot be concluded, and treatment selection should be individualized.

In this case, after treatment with durvalumab (an anti–PD-L1 antibody), pulmonary metastases were detected, and therapy was switched to pembrolizumab (an anti–PD-1 antibody). Recently, cases have been reported in which switching from an anti–PD-L1 antibody to an anti–PD-1 antibody after the development of resistance to the former has resulted in either tumor response or disease stabilization. In a case series of non–small cell lung cancer (NSCLC) by Kitagawa et al., partial responses or stable disease were observed in some patients after rechallenge with an anti–PD-1 antibody following anti–PD-L1 antibody therapy, suggesting that this approach may be a viable treatment option in selected cases (21).

The patient remains alive 26 months after treatment initiation, with no regrowth of the intrahepatic primary tumor up to 18 months after PBT. By contrast, pulmonary and nodal lesions exhibited mild progression, prompting a switch from anti–PD-L1 to anti–PD-1 therapy. Going forward, we will undertake extended follow-up with imaging every 3–4 months, together with clinical and laboratory assessments, and longitudinal evaluation using validated quality-of-life instruments, to comprehensively assess the long-term effectiveness of this treatment plan, in which GCD is administered first, followed by PBT.

Although no treatment-related adverse events were observed in this case, GCD therapy generally carries risks of myelosuppression, nephrotoxicity, and immune-related adverse events (irAEs) (13), whereas PBT may cause radiation-induced liver injury and gastrointestinal/biliary toxicities (7). These risks can be mitigated through baseline and periodic monitoring, adequate hydration with guideline-concordant antiemetic prophylaxis, dose reductions or delays when indicated, early recognition of irAEs with CTCAE-based management, weekly liver-function assessment, and prophylactic acid suppression when high gastrointestinal doses are anticipated (8, 22). Accordingly, a sequential strategy in which GCD therapy is administered first, followed by consolidative PBT, may help balance toxicity reduction with durable local control.

Within the GCD regimen, gemcitabine and cisplatin (GEM/CDDP) have been reported to induce immunogenic cell death (ICD) through tumor cell death and antigen release (23). Meanwhile, chemotherapy and radiation are known to increase tumor PD-L1 expression, and the addition of durvalumab provides a rational strategy to block this therapy-induced immune-suppressive loop (24). Radiotherapy, particularly PBT, can activate antitumor immunity through dendritic cell–mediated cross-priming, forming the basis for both local control and potential abscopal effects (25). Furthermore, PBT concentrates the radiation dose within the tumor while minimizing low-dose exposure to surrounding normal tissues, thereby reducing radiation-induced lymphopenia and preserving systemic immune function (26). These characteristics make PBT highly compatible with immune checkpoint inhibitors. In the present case, ICD induction by GEM/CDDP, enhanced antigen presentation and cross-priming by PBT, and PD-L1 pathway inhibition by durvalumab may have acted synergistically, and together with the MSI-high immune-responsive phenotype, contributed to durable local control and improvement of DIC.

Diverse mechanisms contributing to tumor progression and therapeutic resistance have been reported (27, 28). The assessment of anticancer treatment efficacy has progressed through the use of patient-derived xenograft models in preclinical studies (29). Nevertheless, clinical validation requires real-world evidence derived from larger patient cohorts. As noted in the limitations, this study represents a single case with a limited level of evidence, and therefore caution should be exercised when interpreting and generalizing the findings. This single-case report limits statistical inference and generalizability. To validate the efficacy and universality of the proposed therapy and strengthen the reliability of the conclusions, future studies should secure larger sample sizes to enable robust statistical analyses. When feasible, multicenter prospective registries or comparative trials are recommended.

## 4 Conclusion

This is a clinically valuable case of unresectable iCCA treated with GCD therapy and PBT, resulting in tumor shrinkage, systemic disease control, and durable local control. These findings support the potential of ICI-based chemotherapy combined with PBT as an effective strategy for managing MSI-high iCCA with major vascular invasion.

## Data Availability

The original contributions presented in the study are included in the article/supplementary material. Further inquiries can be directed to the corresponding authors.
